# The role of RAS mutations in leukemia progression, differentiation, and drug resistance

**DOI:** 10.1016/j.lrr.2026.100589

**Published:** 2026-04-11

**Authors:** Congfa Jiang, Hangxuan Wang, Jiaxin Zhao, Yuwei Xu, Shiwei Duan

**Affiliations:** a Department of Hematology, Ningbo Fourth Hospital, Ningbo 315700, Zhejiang, China; b Xiangshan First People's Hospital Medical and Health Group, Ningbo 315700, Zhejiang, China; c Department of Clinical Medicine, School of Medicine, Hangzhou City University, Hangzhou 310015, Zhejiang, China

**Keywords:** RAS mutations, AML, JMML, ALL, Venetoclax resistance

## Abstract

Mutations in the RAS gene family (NRAS, KRAS) are critical drivers of late-stage acute myeloid leukemia (AML) progression. They are frequently detected in relapsed/refractory AML and AML transformed from myelodysplastic syndrome (MDS). Occurring as late-stage genetic events, RAS mutations synergize with early drivers to promote leukemogenesis. While mutually exclusive with FLT3-ITD mutations, they coexist with KIT, RUNX1, CEBPA mutations and MLL rearrangements. Granulocyte-monocyte progenitors (GMPs) serve as the cellular origin for RAS-mutant leukemia stem cells (LSCs). Ultimately, RAS mutations drive monocytic differentiation of LSCs and venetoclax (VEN) resistance through BCL-2 family rewiring. Beyond AML, they are hallmark genetic lesions in juvenile myelomonocytic leukemia (JMML) and present in 15%-20% of pediatric acute lymphoblastic leukemia (ALL) cases. Here, we propose a comprehensive pathogenic model and targeted therapeutic framework focusing on RAS, MCL-1, BCL2L1 to overcome drug resistance and improve patient outcomes.

## 1 Introduction

The RAS gene family (comprising NRAS and KRAS) is among the most frequently mutated oncogene families in human cancers [1]. These mutations have profound implications for the pathogenesis of hematological malignancies, including acute myeloid leukemia (AML), juvenile myelomonocytic leukemia (JMML), and pediatric acute lymphoblastic leukemia (ALL). In AML, RAS mutations are broadly classified as late-acquired genetic events. They demonstrate strong lineage specificity, predominantly clustering in the FAB M4 (myelomonocytic) and M5 (monocytic) subtypes [2,3]. This specific distribution aligns closely with their functional capacity to drive monocytic differentiation [2].

Crucially, RAS mutations alone are insufficient to initiate leukemogenesis [[4], [5], [6]]. Experimental models utilizing engineered human induced pluripotent stem cells (iPSCs) and hematopoietic stem/progenitor cells (HSPCs) confirm that RAS mutations require cooperative synergy with early driver mutations (e.g., ASXL1, SRSF2, TET2, DNMT3A) to induce full malignant transformation. This collaborative pathogenic model is further validated by clinical observations; RAS mutations are frequently gained or lost during disease progression, such as at relapse, confirming their role as secondary, context-dependent regulators of leukemic advancement [7,8].

## 2 Mechanistic insights: cellular origin, pathway activation, and the dual role of RAS mutations

### 2.1 Cellular origin and multistep pathogenic model

**Fig. 1:**
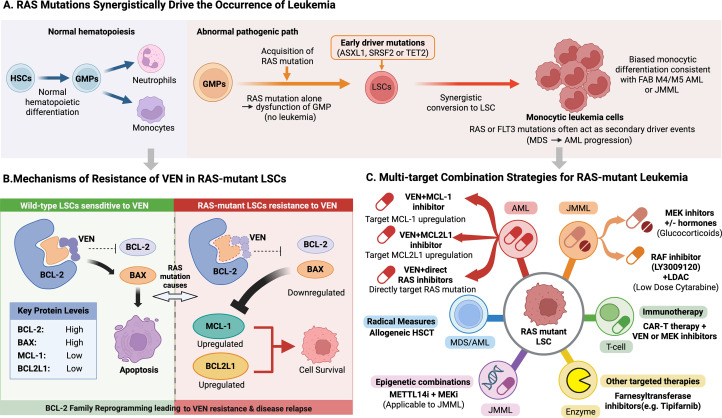
Mechanisms of RAS-driven leukemogenesis and rational combination therapies to overcome VEN resistance A. RAS mutations synergistically drive leukemogenesis. In normal hematopoiesis, hematopoietic stem cells (HSCs) differentiate into granulocyte-monocyte progenitors (GMPs), which further give rise to neutrophils and monocytes. RAS mutations alone cause GMP dysfunction but are insufficient to induce leukemia. When RAS mutations co-occur with early driver mutations (e.g., ASXL1, SRSF2, TET2), they synergistically transform GMPs into leukemia stem cells (LSCs). These LSCs exhibit a monocytic differentiation bias, aligning with the high prevalence of RAS mutations in FAB M4/M5 AML and JMML, ultimately leading to overt disease. Additionally, during MDS-to-AML progression, RAS or FLT3 mutations often act as secondary drivers. B. Mechanisms of VEN Resistance in RAS-mutant LSCs. In RAS-wild-type LSCs, VEN selectively inhibits BCL-2, releasing its suppression on apoptosis and promoting BAX-mediated cell death. In contrast, RAS-mutant LSCs reprogram the BCL-2 family network by downregulating BCL-2 and BAX while upregulating alternative anti-apoptotic proteins such as MCL-1 and BCL2L1 (BCL-XL). This apoptotic rewiring reduces dependency on BCL-2, leading to VEN resistance and disease relapse. C. Multi-target combination strategies for RAS-mutant Leukemia. Proposed therapeutic approaches include targeted therapy such as farnesyl transferase inhibitors (e.g., Tipifarnib); epigenetic combinations such as METTL14 inhibitor plus MEK inhibitor for JMML; VEN resistance reversal via VEN combined with MCL‑1 inhibitor, BCL2L1 inhibitor, or direct RAS inhibitor; immunotherapy such as CAR‑T combined with VEN or MEK inhibitor; and RAS/MAPK pathway targeting through MEK inhibitor plus glucocorticoids or RAF inhibitor LY3009120 plus low‑dose cytarabine.

The cellular context in which RAS mutations emerge heavily dictates their leukemogenic potential [9]. Functional studies identify granulocyte-monocyte progenitor cells (GMPs) as the primary cellular targets for RAS-driven transformation (Fig. 1A) . When RAS mutations are introduced early in hematopoietic development (e.g., at the hematopoietic stem cell stage), the result is GMP dysfunction and aborted leukemogenesis. Conversely, when RAS mutations arise in GMPs that already harbor early driver mutations (such as ASXL1 or SRSF2), they facilitate the conversion of GMPs into leukemia stem cells (LSCs). This occurs via extensive remodeling of the transcriptomic and chromatin landscapes, directly accounting for the enrichment of RAS mutations in FAB M4/M5 AML [9].

Leukemogenesis driven by RAS mutations is a highly cooperative, multistep cascade beginning with initiation through the acquisition of early driver mutations such as ASXL1 and SRSF2. Progression follows with the subsequent acquisition of RAS mutations, which are often mutually exclusive with FLT3-ITD [6,7]. Transformation occurs as early and late events synergize to convert GMPs into LSCs. This process leads to differentiation in which RAS mutant LSCs skew toward monocytic differentiation, resulting in FAB M4/M5 AML or JMML [2,10,11]. Finally, resistance emerges following disease onset and venetoclax (VEN) treatment, as selective pressure induces acquired resistance in RAS mutant cells via BCL 2 family protein reprogramming, culminating in relapse.

### 2.2 Pathway activation and dual role in differentiation and resistance

Hyperactivation of the RAS pathway is not exclusively driven by NRAS/KRAS point mutations . Alternative activating mechanisms include BRAF mutations (which directly stimulate downstream signaling) [12], METTL14-mediated m⁶A RNA modification (which sustains RAS-driven proliferation by regulating autophagy) [13], and the constitutive activation of the MAPK/ERK pathway, RAS's primary downstream effector [14,15]. Recognizing these non-canonical mechanisms expands the definition of "RAS-driven" leukemias and highlights the critical need for broad, pathway-targeted therapies [[12], [13], [14], [15]].

RAS mutations play a context-dependent, dual role in leukemia: acting simultaneously as differentiation drivers and mediators of drug resistance [16]. Intrinsically, they bias LSC fate toward the monocytic lineage, which clinically correlates with poor responses to VEN. This is evidenced by elevated bone marrow monocyte counts in RAS-mutant AML patients, the upregulation of monocyte-specific markers (CD14, CD64) in iPSC-derived LSCs, and the observation that RAS mutations promote GMP monocytic differentiation across varied genetic backgrounds [2,9].

Extrinsically, they directly mediate VEN resistance. Venetoclax, a selective BCL-2 inhibitor and standard-of-care for AML [17], faces primary resistance in 20%–30% of patients and drives relapse in over 40% of initial responders. RAS mutations dramatically exacerbate this clinical challenge: patients with N/KRAS-mutant AML treated with VEN and hypomethylating agents (HMAs) suffer higher relapse rates and shorter overall survival. The fundamental mechanism of this resistance is the reprogramming of the apoptotic machinery within RAS-mutant LSCs. This features the downregulation of VEN targets (BCL-2/BAX) paired with the compensatory upregulation of alternative anti-apoptotic proteins like MCL-1 and BCL2L1/BCL-XL (Fig. 1B) [16,18].

## 3 Rational therapeutic framework for RAS-mutant leukemias

Guided by this mechanistic evidence, we propose a personalized therapeutic framework for RAS-mutant leukemias (Fig. 1C), anchored by upfront RAS mutation screening to properly stratify treatment [8].

To reverse VEN resistance, rational combination strategies must restore a lethal balance among BCL 2 family proteins [16]. Promising approaches include VEN plus MCL 1 inhibitors such as S64315, which block the primary upregulated survival protein in RAS mutant cells. Clinical trials are currently evaluating this synergy, as seen in NCT03672695. Another strategy is VEN plus BCL2L1 inhibitors like Navitoclax, targeting a secondary resistance mediator, though this requires careful dosing to mitigate toxicities such as thrombocytopenia [19]. Additionally, VEN plus direct RAS inhibitors such as AMG 510 for KRAS G12C can reduce upstream pathway activation to consequently downregulate MCL 1 and BCL2L1 expression [16].

Directly targeting the RAS pathway also enhances anti-leukemic efficacy [18,20]. Validated preclinical strategies include pairing MEK inhibitors (e.g., Trametinib) with glucocorticoids to force apoptosis in LSCs [18], and combining the RAF inhibitor LY3009120 with low-dose cytarabine (Ara-C) to suppress AML proliferation [20]. For JMML, combining METTL14 inhibitors with MEK inhibitors effectively neutralizes both epigenetic and signaling dependencies [13].

Alternative and curative interventions include Farnesyl Transferase Inhibitors (FTIs) like Tipifarnib [21,22]; allogeneic hematopoietic stem cell transplantation (HSCT), which leverages the susceptibility of RAS-mutant LSCs to graft-versus-leukemia (GVL) effects [10,23]; and intensive chemotherapy with high-dose Ara-C [11,24]. Furthermore, integrating immunotherapy such as combining Chimeric Antigen Receptor T cell (CAR-T) therapy with VEN or MEK inhibitors offers significant promise [25]. VEN can prime LSCs for CAR-T-mediated cell death, while MEK inhibitors may suppress the immune evasion tactics of the tumor [25].

## 4 Discussion

**Fig. 2:**
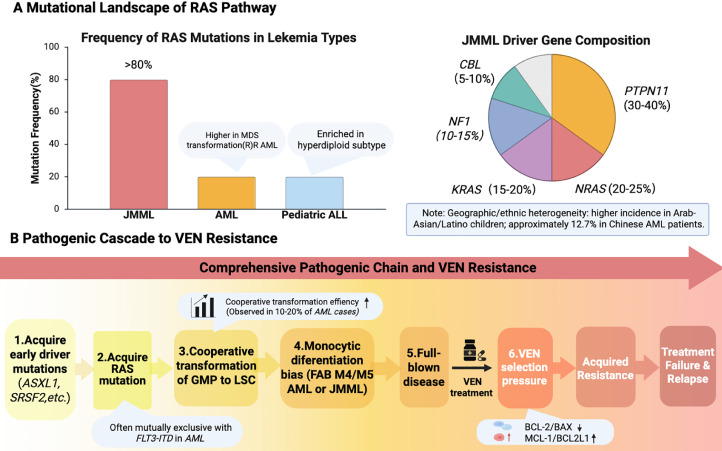
Epidemiological landscape and integrated pathogenic model of RAS mutations in leukemia A. Mutational Landscape of RAS Pathway. (Left) Bar graph depicting the frequency of RAS pathway mutations in three major leukemia types. Juvenile myelomonocytic leukemia (JMML) shows the highest mutation frequency (>80%), driven by multiple pathway genes (PTPN11, NRAS, KRAS, NF1, CBL), as detailed in the right pie chart. In AML, the overall mutation frequency is 10%–20%, with higher rates in AML secondary to MDS or relapsed/refractory disease. In pediatric ALL, mutations occur in 15%–20% of cases, particularly enriched in hyperdiploid subtypes. Notably, RAS mutation frequencies exhibit geographic and ethnic heterogeneity. B. Pathogenic cascade leading to VEN Resistance. The flowchart outlines the multistep leukemogenesis process, beginning with the acquisition of early driver mutations such as ASXL1 and SRSF2, followed by the subsequent acquisition of RAS mutations, which are often mutually exclusive with FLT3-ITD in AML. These early and late events cooperate synergistically to transform GMPs into LSCs. RAS-mutant LSCs then preferentially differentiate toward the monocytic lineage, leading to FAB M4/M5 AML or JMML and culminating in full disease manifestation. Finally, VEN treatment imposes selective pressure that drives acquired resistance in RAS-mutant cells through BCL-2 family reprogramming, characterized by downregulation of BCL-2/BAX and upregulation of MCL-1/BCL2L1, ultimately resulting in treatment failure and relapse.

RAS pathway mutations display highly distinct distribution patterns across leukemia subtypes (Fig. 2A) [1,8,19]. JMML exhibits the highest frequency (>80%), driven by an array of pathway genes including PTPN11, NRAS, KRAS, NF1, and CBL [10,11,26,27]. In AML, the baseline frequency is 10%–20%, though this spikes in AML secondary to MDS or in relapsed/refractory cases [[6], [7], [8]]. Pediatric ALL sees a 15%–20% frequency, largely enriched within hyperdiploid subtypes [28].

Notably, the prevalence of RAS mutations is subject to significant geographic and ethnic variability [3,19]. Higher rates are observed in Arab-Asian and Latino pediatric cohorts [3,19], contrasting with lower rates in Chinese AML populations (∼12.7%), where RAS status does not always serve as an independent prognostic factor [4,5]. This heterogeneity mirrors the tissue-specific oncogenic contexts seen in solid tumors like pancreatic adenocarcinoma [1] and reinforces the multistep nature of RAS-driven leukemogenesis.

This review expands standard models of leukemic progression by re-defining RAS mutations as strictly context-dependent drivers [8,9]. Their clinical impact requires subtype-specific precision medicine (Table 1). For instance, PTPN11 mutations correlate with the poorest prognosis in JMML, making MEK inhibitors a priority [26,27]. In pediatric ALL, RAS mutations worsen outcomes primarily in high-risk BCR-ABL1-like subtypes [19,28]. Future success relies on multi-targeted strategies and ensuring diverse patient cohorts in clinical trials to confirm global applicability [3,19].

**Table 1:** Proposed targeted therapy strategies for RAS-mutant leukemias.

Strategy Category	Target/Agent Example	Potential Leukemia Context	Rationale / Proposed Mechanism	Supporting Evidence
**Overcome VEN Resistance**	VEN + MCL-1 inhibitor	AML (RAS-mutant)	Counteract MCL-1 upregulation to restore apoptotic sensitivity	[19]
	VEN + BCL2L1 inhibitor	AML (RAS-mutant	Counteract BCL2L1 (BCL-XL) upregulation to restore apoptotic sensitivity	[19]
	VEN + direct RASi	AML (RAS-mutant	Target the upstream oncogenic driver	[16]
**MAPK Pathway Inhibition**	MEK inhibitor (Trametinib ± Glucocorticoids	AML, JMML	Block RAS downstream signaling to impair leukemic cell survival and proliferation	[18]
	RAF inhibitor (LY3009120 + LDAC	AML (RAS-mutant)	Synergistic cytotoxicity through concurrent pathway inhibition	[20]
**^Epigenetic Combination^**	METTL14i + MEKi	JMML	Dual targeting of m6A-modification-mediated autophagy and MAPK signaling	[13]
**^Other Targeted Therapy^**	Farnesyl Transferase Inhibitor (e.g., Tipifarnib)	AML, MDS	Alternative post-translational inhibition of the RAS pathway	[21,22]
**^Curative Modality^**	Allogeneic HSCT	JMML, high-risk AML	Potentially curative immunotherapy; standard for JMML	[10,23]
**Immunotherapy Combination**	CAR-T + VEN/MEKi	R/R AML	Synergy between immune-mediated cytotoxicity and targeted pathway/apoptosis induction	[25]

Strategies are categorized by their primary mechanistic approach. Abbreviations: VEN, venetoclax; LDAC, low-dose cytarabine; HSCT, hematopoietic stem cell transplantation; R/R, relapsed/refractory. Note: The strategy "VEN + direct RASi" is proposed based on the core pathogenic mechanism but requires further clinical validation.

## 5 Limitations and future directions

Our analysis acknowledges several limitations. Many foundational clinical studies are retrospective and feature small sample sizes, which inherently limits statistical power [7,8]. The lack of long-term follow-up in novel combination trials precludes a definitive assessment of treatment durability [16,21]. Furthermore, focusing on single-agent resistance fails to capture the complexity of modern multi-drug regimens; the interplay between RAS mutations, broad multi-drug resistance, and epigenetic crosstalk (e.g., ASXL1, TET2) remains poorly mapped [13,16]. Lastly, how RAS mutations alter the immune microenvironment to facilitate immune evasion requires much deeper exploration [16].

Future research should prioritize validating combination therapies in large, prospective, multi center cohorts [17,19]. Long term efficacy and safety of regimens such as VEN plus MEK inhibitors need to be tracked [20,21]. Researchers must also focus on deciphering the intersection of RAS signaling, epigenetic regulation, and immune evasion to expose new vulnerabilities [9,13]. Additionally, standardizing sensitive detection methodologies, including NGS and ddPCR, is essential to actively guide clinical decisions[8].

## 6 Conclusion

By illuminating the dual role of RAS mutations as both differentiation drivers and resistance mediators, this framework substantially advances our understanding of RAS-mutant leukemias [1,16]. It provides a rigorous mechanistic basis for molecular subtyping, supports rational treatment selection (e.g., VEN + MCL-1 inhibitors, Fig. 1C), and underscores the urgent need for subtype-specific interventions. Through sustained, multidisciplinary collaboration and diverse global clinical trials, the individualized strategies proposed here can transition into standard clinical practice, ultimately improving survival outcomes for patients battling these aggressive hematological malignancies [16].

## List of abbreviations

AML, acute myeloid leukemia;

ALL, acute lymphoblastic leukemia;

Ara-C, Cytarabine;

BCL2L1, BCL2 Like 1;

CAR-T, Chimeric Antigen Receptor T;

CEBPA, CCAAT Enhancer Binding Protein Alpha; ddPCR, droplet digital polymerase chain reaction;

DNMT3A, DNA Methyltransferase 3 Alpha;

FDA, Food and Drug Administration;

FAB, French-American-British classification;

FTI, Farnesyl Transferase Inhibitor;

GMPs, Granulocyte-Monocyte Progenitor Cells;

HSCT, Hematopoietic Stem Cell Transplantation;

HSPC, Hematopoietic Stem/Progenitor Cells; iPS, Induced Pluripotent Stem;

JMML, Juvenile Myelomonocytic Leukemia;

LDAC, low-dose cytarabine; LSCs, Leukemia Stem Cells;

MCL1, Myeloid Cell Leukemia 1;

MEK, Mitogen-Activated Protein Kinase;

MDS, Myelodysplastic Syndrome; m6A, N6-Methyladenosine;

NGS, next-generation sequencing;

RAF, Rapidly Accelerated Fibrosarcoma;

RASi, RAS Inhibitors;

TET2, Ten-Eleven Translocation 2;

VEN, Venetoclax.

## Ethics approval and consent to participate

Not applicable.

## Consent for publication

All authors have read and agreed to the published version of the manuscript.

## Availability of data and material

Not applicable.

## Funding source

This study was supported by the Qiantang Scholars Fund in Hangzhou City University (No. 210,000–581,835).

## CRediT authorship contribution statement

**Congfa Jiang:** Conceptualization, Visualization, Writing – original draft. **Hangxuan Wang:** Conceptualization, Visualization, Writing – original draft. **Jiaxin Zhao:** Conceptualization, Visualization. **Yuwei Xu:** Conceptualization, Visualization. **Shiwei Duan:** Conceptualization, Funding acquisition, Supervision, Writing – review & editing.

## Declaration of competing interest

The authors report no competing interests.
